# Voltammetric Determination of Cyproterone Acetate in Pharmaceutical Preparations

**Published:** 2010-06

**Authors:** Nahed El-Enany, Dina El-Sherbiny, Fathalla Belal

**Affiliations:** 1 *Department of Analytical Chemistry, Faculty of Pharmacy, Mansoura University, Mansoura, Egypt;*; 2 *Department of Medicinal Chemistry, Faculty of Pharmacy, Mansoura University, Mansoura, Egypt*

**Keywords:** cyproterone acetate, Direct Current time controlled (DC_t_), differential pulse (DPP), pharmaceutical dosage forms

## Abstract

The voltammetric behaviour of cyproterone acetate (CPA) was studied using direct current (DC_t_) and differential pulse polarography (DPP). The drug manifests cathodic waves over the pH range of 4-11.8. In Britton-Robinson buffer (BRb) of pH 10, the diffusion current-concentration relationship was found to be rectilinear over the range 3.2-32 μg/mL and 0.5-14 μg/mL using DC_t_ and DPP modes, respectively, with minimum limits of detection (LOD) of 0.13 μg/mL using the DDP. The diffusion-current constant (Id) was 9.29 ± 0.046 (n=9). The proposed method was successfully applied to the determination of the studied compound in its formulations. The mean percentage recoveries in tablets were 99.48 ± 1.25 and 100.01 ± 1.07 (n=4) using DC_t_ and DPP modes, respectively. The results obtained were in agreement with those of the reference method. A proposal for the electrode reaction was postulated.

## INTRODUCTION

**Figure 1:**
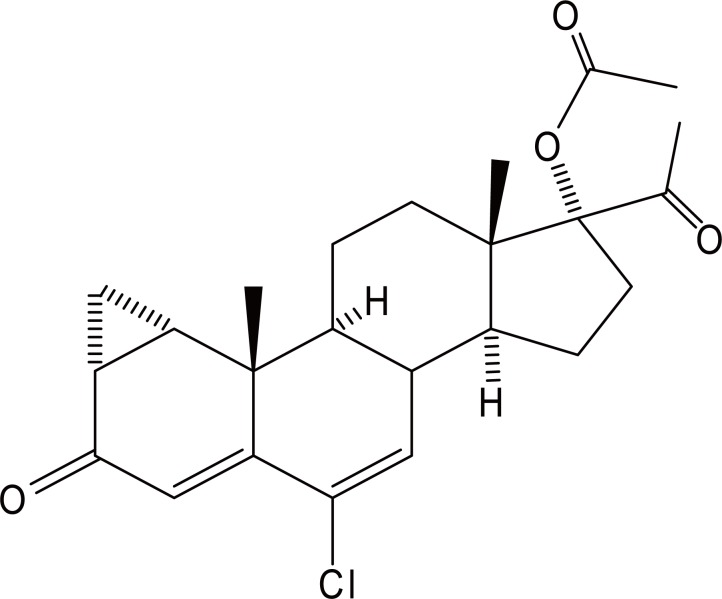
Structural Formula of cyproterone acetate.

Cyproterone acetate (CPA), 6-chloro-1β,2β-dihydro-17-hydroxy-3′*H*-cyclopropa[1, 2]pregna-4,6-diene-3,20-dione 17- acetate (Fig. 1) is a progestogen with antianddrogenic properties. It is used for the control of libido in severe hypersexuality or sexual deviation in men. It is also used for the palliative treatment of prostatic carcinoma. CPA is used with ethinylestradiol in woman for the control of acne and hirsutism and provides also contraception in those women (1).

Literature survey reveals some methods for the determination of CPA in pharmaceutical preparations including spectrophotometry (2-4), HPLC (5-8) and GC-MS (9). Although capillary electrophoresis offers minute sample volume and short analysis time but it is highly sophisticated technique which may not be available in quality control laboratories. Therefore, there is a need for an alternative substitute to these techniques for the routine quality control analysis of CPA, and polarography was a promising substitute. Review of literature revealed that, up to the present time, there have been no reports concerning the electrochemical behavior of CPA. The molecular structure of cyproterone acetate reveals the presence of carbonyl group conjugated with a double bond, which initiated the present study. A simple, specific and sensitive method was developed for the determination of cyproterone in its dosage forms, based on the reduction of the keto group into the corresponding hydroxyl, at the dropping mercury electrode (DME).

## EXPERIMENTAL

### Apparatus

The polarographic study, DC_t_ and the DPP measurements were carried out using the Polarecord E 506 Metrohm (Herisau, Switzerland). The mercury drop-time of 1 sec was electronically controlled using the 663 VA Stand from the same company. The polarograms were recorded using a potential scan rate of 10 mV/sec. A three-electrode electrochemical cell comprising a Dropping Mercury Electrode (DME) as the working electrode, an Ag/AgCl reference electrode, and a graphite rod as the auxiliary electrode, was used. Phase selective AC_t_ polarograms were recorded using the same instrument; the superimposed alternating voltage being 15 mV at a frequency of 75 Hz and a phase angle of 90°.

### Materials and reagents

All materials and reagents used were of analytical reagent grade.

- Cyproterone acetate reference standard was kindly supplied by Glaxo SmithKline.
- Tablets containing Cyproterone acetate (Diane-35^®^ tablets labeled to contain 2 mg Cyproterone acetate +0.035 mg ethynylestradiol per tablet) (Batch # 502 B) were purchased from a local pharmacy.
- Britton-Robinson Buffers (BRb): 0.08 M solution covering the pH range 4.0-11.8 (a mixture of each of acetic, orthophosphoric and boric acids, adjusted to the required pH with 0.4M sodium hydroxide (10).
- Methanol (Sigma, St. Louis, MO, USA).

A stock solution containing 100 μg/mL of cyproterone acetate was prepared in methanol, and further diluted with the same solvent as appropriate. The stock solutions were stable for 10 days when kept in the refrigerator.

## GENERAL PROCEDURE

Aliquots of the stock solution were transferred into a set of 25 mL volumetric flasks so that the final concentration is in the range of 3.2-32 and 0.5-14 μg/mL for the DC_t_ and DPP modes, respectively. The solution was completed to the volume with BRb of pH10.0. The whole contents of the flasks were transfered into the polarographic cell, nitrogen gas was passed for 5 min, then the polarograms were recorded in both the DC_t_ and DP polarographic modes respectively over the potential range -0.8 to -1.6 V *versus* Ag/AgCl. The current (μA) of each of DC_t_ or DPP were plotted *versus* the concentration (μg/mL) to get the calibration graphs. Alternatively, the corresponding regression equations were derived.

### Determination of Cyproterone acetate in Diane-35™ tablets

Ten tablets were weighed and pulverized well. A weighed quantity of the powder equivalent to 10.0 mg of the drug was transfered into a small conical flask. The drug was extracted three times each with 30 mL of methanol, the extracts were filtered through Whatman filter paper into a 100 mL volumetric flask. The conical flask was washed with few mls of methanol and the washings were passed into the same volumetric flask. Then, the solution was completed to the volume with methanol. Aliquots containing the working concentration range was transferred into 25 mL volumetric flasks. Complete as described under “*General procedure*”. The nominal content of the tablets (concentration found) was determined either from the calibration graph or using the corresponding regression equations adopting either of the DC_t_ or DPP modes.

## DISCUSSION

**Figure 2:**
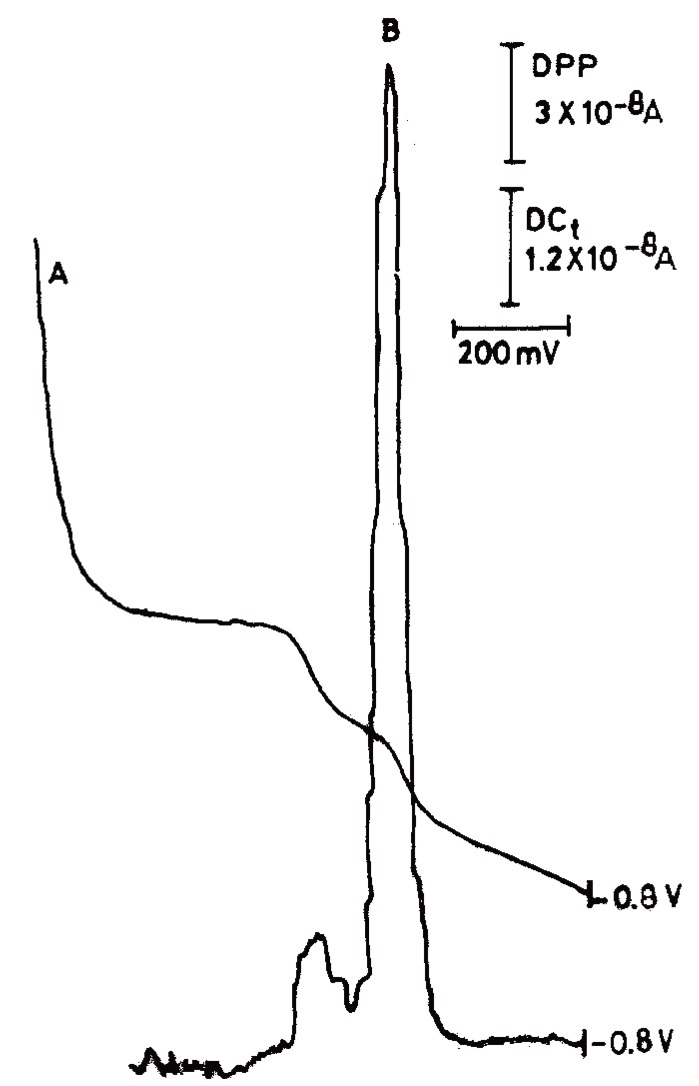
Typical DC_t_ (curve A) and DPP (curve B) polarograms of cyproterone acetate (10 μg/ mL) in BRb of pH 10.0. A, DC_t_ mode; B, DPP mode, respectively.

**Figure 3:**
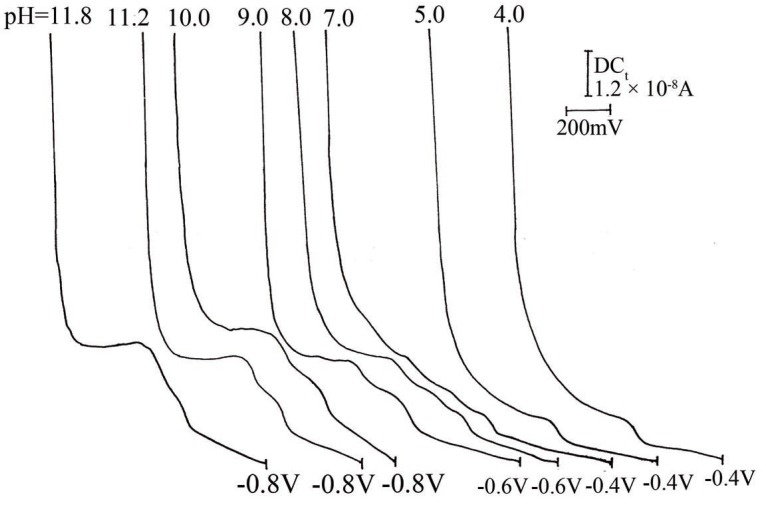
Effect of pH on the development of the polarographic waves of cyproterone acetate (10 μg/ mL).

**Figure 4:**
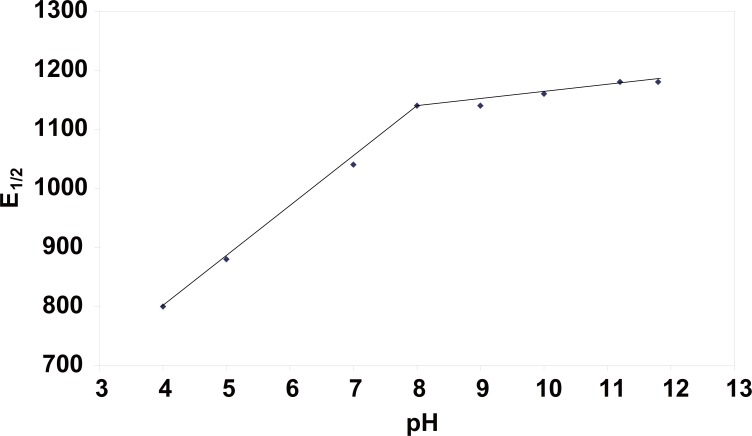
Effect of pH on E_½_ (mV) values of cyproterone acetate (10 μg/ml) in BRb containing 20 % methanol.

Figure 2 shows the typical (DC_t_ and DPP) polarograms of CPA in BRb of pH10.0 containing 20% methanol. Methanol was added as a solubilizer for CPA, it also decreased the adsorption of the drug likely to occur at the surface of DME. Cyproterone acetate produces well-defined cathodic waves over the pH range of 4-11.8 in BRb (Fig. 3). Reduction of CPA at the dropping mercury electrode was found to be pH dependent, as the E_½_ values were shifted to more negative values upon increasing the pH (Fig. 3). A plot of E_½_ *versus* pH gave two straight lines with one break at pH8 (Fig. 4). The relation between E_½_ values and the pH of the solution is represented by the following equations:

E_½_ = 480 + 80 pH      (r=1.000)

over the pH range 4-7 and

E_½_ = 1050.4 + 11.36 pH    (r=0.973)

over the pH range 8-11.8

The number of electron transfer at the rate determining step (αn_a_) were calculated from the equation of Meites and Israel (11).

E= E_½_- (0.059/ αn_a_) log[i/id-i]

where id is the diffusion current and α is the transfer coefficient. Logarithmic analysis of the reduction waves obtained in BRb of different pH values resulted in straight lines. The αn_a_ values were calculated according to the treatment of Meites and Israel (11) and are listed in Table 1, at pH10.0, αn_a_ was 0.885. Assuming that the rate-determining step involves the transfer of two electrons, the value of αn_a_ point out to the completely irreversible nature of the reduction process.

**Table 1:** Effect of pH on the development of the polarographic waves of cyprotrone acetate

pH	-E_½_ (mV)	-Δ E_½_ / ΔpH	W_½_ (mV)	αn_a_

4.0	800	80.0	50	0.66
5.0	880	90.0	40	0.738
7.0	1040	100.0	50	0.404
8.0	1140	10.0	60	0.450
9.0	1150	20.0	35	0.501
10.0	1170	8.3	30	0.885
11.2	1180		50	0.786
11.8	1180		60	0.710

W_½_, Half-peak width in the DPP mode; n_a_: Number of electrons transferred in the rate-determining step; α: Transfer coefficient.

### Study of the wave characteristics

Changing the buffer concentration over the range 6 × 10^-3^ M to 6 × 10^-2^ M was found to yield a negligible effect on the wave height of CPA. This indicates a diffusion-controlled wave, partially affected by adsorption phenomenon.

Cyproterone acetate was found to be stable in BRb of pH10.0 (the analytical pH) for about one and half hour at room temperature, after which the peak height began to decrease slowly.

The diffusion current constant (Id) was calculated according to Ilkovic equation (12) for varying concentrations of the drug using the following equation:

Id = id/ C m^2/3^ t^1/6^

and was found to be 9.29 ± 0.046 (n=9). The results are shown in Table 2.

**Table 2:** Correlation between the concentration of CPA and the diffusion current in the DC_t_ mode

No	Concentration (mM)	Current (μA)	id/C (μA/mM)	Id = id/Cm^2/3^t^1/6^

1	0.00768	0.0864	11.25	9.282
2	0.0096	0.1080	11.25	9.282
3	0.0192	0.2160	11.25	9.282
4	0.0240	0.2730	11.375	9.385
5	0.0288	0.3240	11.25	9.282
6	0.0384	0.4290	11.172	9.218
7	0.0480	0.540	11.25	9.282
8	0.0576	0.650	11.28	9.307
9	0.0768	0.8610	11.211	9.250
Mean				9.29
± SD				± 0.046

Each result is the average of three separate determinations. Id, Limiting diffusion current constant; id, Limiting diffusion current (μA); C, Concentration in m mole/L; M, Flow rate in mg/ sec; T, Drop time in second.

### Mechanism of electrode reaction

**Figure 5:**
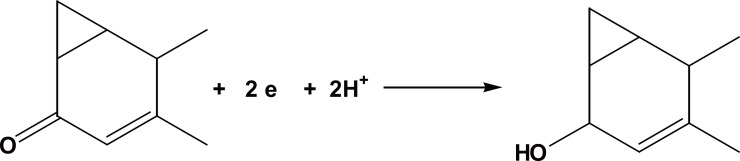
Postulated pathway.

The number of electrons consumed during the reduction process was accomplished through comparison of the waveheight of cyproterone with that obtained from an equimolar solution of a previously studied, structurally related, compound and of nearly identical value of diffusion coefficient, namely Spironolactone (13). In BRb of pH10.0, both compounds gave one wave, of the same height. Hence, it is concluded that 2 electrons are involved in the reduction process. Based on the presence of carbonyl group, and by analogy to the reported mechanism of reduction proposed for Spironolactone, the following pathway is postulated (Figure 5).

### Analytical performance

Under the described polarographic conditions, at pH10.0, CPA exhibits a well defined diffusion controlled cathodic wave and sharp differential pulse peak, both are suitable for analytical applications. No polarographic maximum was developed; hence no maximum suppressor was needed.

Plots representing the relationship between the diffusion current of both the DC_t_ and DPP modes versus the concentration of CPA gave straight lines over the concentration ranges of 3.2-32 and 0.5-14 μg/ml using DC_t_ and DPP modes respectively, with minimum limits of detection (LOD) of 0.15 and 0.13 μg.ml^-1^ using DC_t_ and DPP modes, respectively (Table 3).

Linear regression analysis of the data gave the following equations:

id = 8.97 × 10^-4^ + 0.027 C     (r=0.9999)

using DC_t_ mode....... and

ip = 1.355 × 10^-3^ + 0.026 C       (r=0.9999)

using DPP mode.

where C is the concentration in μg/ml, id is the diffusion current in μA in the DC_t_ mode and ip is the current in μA in the DPP mode.

**Table 3:** Analytical parameters for the polarographic determination of cyproterone acetate using DC_t_ and DPP modes

Parameter	DC_t_ mode	DPP mode	Reference Method (2)

No. of experiments	9	9	3
Mean found (%) ± SD	99.96 ± 0.61	100.02 ± 0.94	100.49 ± 0.49
Variance	0.372	0.24	
Student’s *t*-value	1.37 (2.23)	0.81 (2.23)	0.24
Variance ratio F-test	1.55 (4.46)	3.68 (4.46)	
Concentration range (μg/ml)	3.2-32	0.5-14	
Limit of detection (LOD) (μg/ml)	0.15	0.13	
Correlation coefficient (r)	0.9999	0.9999	
Intercept	8.97 × 10^-4^	1.36 × 10^-3^	
Slope	0.027	0.026	
S_y/x_	2.03 × 10^-3^	1.47 × 10^-3^	
S_a_.	1.25 × 10^-3^	9.67 × 10^-4^	
S_b_.	7.48 × 10^-5^	1.23 × 10^-4^	
% Error	0.20	0.31	

Values between parentheses are the tabulated *t* and F values respectively, at *p*=0.05 (15). S_y/x_, standard deviation of the residual; S_a_, standard deviation of the intercept of the regression line; S_b_, standard deviation of the slope of the regression line; %Error, %RSD/√n.

### Method validation

The proposed method was validated using the following criteria; linearity, sensitivity, intra-day and inter-day precision, accuracy, specificity, and robustness.

### Linearity

Linearity was evaluated by calculation of the regression equations over the ranges given in Table 3.

The sensitivity of the method was evaluated by determining the limit of detection (LOD) according to ICH Q2B guidelines (14).

LOD=3.3 S_a_/b

where the standard deviation of the intercept of the regression lines and b=the slope of the calibration curve. Statistical evaluation of the regression lines regarding standard deviation of the residual (S_y/x_), standard deviation of the intercept (S_a_) and standard deviation of the slope (S_b_) is given in Table 3. The small value of the figures indicates the high accuracy and high precision of the method (15).

### Accuracy

To test the validity of the proposed method it was applied to the determination of authentic sample of CPA over the concentration range cited in Table 3. The results obtained were in good agreement with those obtained using a reference UV derivative spectrophotometric method (2). Using Student’s t-test and variance ratio F-test (15) revealed no significant difference between the performance of the two methods regarding the accuracy and precision, respectively (Table 3).

### Precision

**Repeatability.** The repeatability was performed through analysis of two concentrations of CPA in pure forms adopting the two polarographic modes (DPP and DC_t_) on three successive times, and the results are listed in Table 4.

**Table 4:** Validation of the proposed method for the determination of cyproterone acetate in pure form

Precision	DC_t_ mode		DPP mode	
	10 μg/ml	20 μg/ml	10 μg/ml	14 μg/ml

Repeatability	99.63	100.93	101.16	100.11
	100.04	101.31	99.61	100.67
	100.75	100.75	99.42	99.55
Mean	100.14	100.99	100.06	100.11
± S.D.	0.57	0.29	0.95	0.56
% RSD	0.57	0.29	0.95	0.56
% Error	0.33	0.17	0.55	0.32
Intermediate precision	101.88	101.89	100.77	100.40
	100.38	99.45	99.23	99.27
	99.81	100.37	98.84	100.14
Mean	100.69	100.37	99.61	99.94
± S.D.	1.06	1.23	1.02	0.59
% RSD	1.06	1.23	1.02	0.59
% Error	0.61	0.71	0.59	0.34

Intermediate precision. It was performed through repeated analysis of CPA in pure form applying the proposed method, using the concentrations showed in Table 4, for a period of three successive days.

The repeatability and reproducibility in both modes were fairly good, as indicated by the small values of standard deviation (SD), relative standard deviation (RSD), and percentage error (% Er).

### Robustness

The robustness of the method is demonstrated by the consistency of the diffusion current with minor changes in experimental variables that might reasonably be expected to take place during the course of the operation of the method, such as changing the molar concentration of the buffer over the range of 0.006-0.06 M resulted in a negligible effect on the wave height of CPA.

### Applications

Both DC_t_ and DPP modes were successfully applied to the assay of CPA in its commercial tablets (Diane-35™). The percentage recovery based on 4 separate determinations are abridged in Table 5. The results are in agreement with those obtained using a reference UV derivative spectrophotometric method (2), where the first derivative of the methanolic solution was measured at 303 nm. Statistical analysis of the results using the Student’s *t*-test and the variance ratio F-test (15) revealed no significant difference between the performance of the two methods regarding accuracy and precision, respectively (Table 5).

**Table 5:** Polarographic determination of cyproterone acetate in its tablets using the proposed and the reference methods

Pharmaceutical preparation	DC_t_ mode		DPP mode		ReferenceMethod (2) % Recovery
	Labeled amount (mg)	% recovery	Labeled amount (mg)	% recovery	

Diane-35™ tablets^a^	2	101.33	2	98.99	100.54
(cyproterone acetate 2mg + 0.035 mg ethynyestradiol/tablet)	2	98.61	2	99.19	99.43
	2	98.90	2	100.91	101.59
(Batch # 502 B)	2	99.07	2	100.96	
Mean		99.48		100.01	100.52
± SD.		1.25		1.07	1.08
Student’s t-value		1.15		0.62	
Variance ratio F-test.		1.34		1.02	

The tabulated values of t and F are (2.57) and (9.55) respectively, at *p*=0.05 (15).

a product of Schering AG Germany.

### Specificity

The specificity of the method was investigated by observing any interference encountered from the common excipients, such as talc, lactose, magnesium stearate, avisil and starch. These excipients did not interfere with the proposed methods.

## CONCLUSION

A simple and sensitive method was developed for the determination of CPA in formulations. It has distinct advantages over other existing methods regarding sensitivity, saving time. Moreover, the proposed method does not require elaborate treatment for the sample or prior extraction for pure form. As well as, the method is sensitive enough for the analysis of lower concentration of CPA as low as 0.5 μg/ml.
